# Suppressive effects of valproic acid on caudal fin regeneration in adult zebrafish

**DOI:** 10.1080/19768354.2020.1860126

**Published:** 2020-12-24

**Authors:** Yunkyoung Lee, Dohee Kim, Chang-Joong Lee

**Affiliations:** Department of Biological Sciences, Inha University, Incheon, Korea

**Keywords:** zebrafish, valproic acid, regeneration, caudal fin, BrdU

## Abstract

Zebrafish can regenerate fins following injury through an epimorphic process that includes the formation of new tissues and reconstruction of the original morphology. In this study, the effects of valproic acid (VPA), a widely used anti-epileptic drug, on fin regeneration were studied after the caudal fin amputation of adult zebrafish. In the control group, zebrafish formed new tissues and reconstructed the original rays 14 days after amputation (dpa). Meanwhile, VPA treatments between 20 and 200 µM following amputation suppressed fin regeneration in a dose-dependent manner and altered morphological characteristics, such as bifurcation and segmentation, in the rays. Compared to the control, VPA also delayed blastema formation and decreased cell proliferation in the mesenchymal area of the regenerated fin. The mRNA expression of *lef1,* a downstream signaling gene in the Wnt pathway, was transiently increased in the regenerated fin of the control at 2 dpa; the same increase was not observed in the VPA-treated zebrafish. Sodium butyrate (SB), an histone deacetylase activity (HDAC) inhibitor, suppressed the fin regeneration without affecting the morphological characteristics of the regenerated ray. Furthermore, the transient increase of *lef1* mRNA was not suppressed in the SB-treated zebrafish. These results suggested that VPA's suppressive effects on fin regeneration are partly mediated through decreased cell proliferation and *lef1* mRNA expression.

## Introduction

Zebrafish have been extensively used to study the regenerative mechanism and investigate pharmacological agents affecting the regeneration processes in adult animals. In particular, the caudal fin of zebrafish, which consists of the dorsal and ventral lobe with 15–18 rays per each lobe, has shown a distinct anatomical morphology and regenerative capabilities (Becerra et al. 1983). Each ray, also known as lepidotrichia, is an endoskeleton with a series of segments. A lepidotrichia consists of two hemirays that face each other. Most rays have a bifurcation; a stretched ray can be split into two sister rays. After amputation, fin regrowth results in new distal segments and reconstructs the original length of fin rays (Shibata et al. 2018). Fin regeneration occurs in three stages, wound healing, blastema formation, and outgrowth (Pfefferli and Jaźwińska 2015). During the wound healing stage, cells around the damaged region migrate to make a thin epidermis. During the blastema formation stage, a blastema, a proliferative mass of mesenchymal cells located at the tip of each ray (Santos-Ruiz et al. 2002), is developed under the newly formed epidermis. Finally, the cells in the blastema proliferate and differentiate into new tissues, eventually reconstructing the original morphology. While regeneration and development undergo different pathways, they appear to have similar aspects. Therefore, it is of interest to study whether chemicals that affect the developmental processes also influence the regenerative capabilities in adult animals.

Valproic acid (VPA), a widely used pharmacological compound for treating convulsive and affective disorders (Chiu et al. 2013; Romoli et al. 2019), is also well-known for its teratogenic effects during development in both human and experimental animals (Ornoy 2009). Generally, the teratogenic effect on embryos and fetuses induced by VPA can be manifested in two aspects. First, it induces congenital anomalies in various organs, including the heart, cleft, limbs, and brain. Facial and skeletal malformation have been frequently reported in the offspring whose mothers are exposed to VPA during pregnancy (Mutlu-Albayrak et al. 2017). Deterred limb growth, hypoplasia, is one of the abnormalities caused by the maternal use of VPA (Rodríguez-Pinilla et al. 2000). Experimental animals exposed to VPA during development have shown serious neural, craniofacial, and skeletal birth defects (Okada et al. 2009). Fused vertebrae and ribs, supernumerary ribs, and an extra pair of vertebrosternal ribs were observed in VPA-treated mice at the embryonic stage (Okada et al. 2009). Second, VPA even affects cognitive function and behavior. The children exposed to VPA during pregnancy have reduced language abilities and often have autistic behaviors (Nicolini and Fahnestock 2018).

A few studies have demonstrated that VPA inhibits limb regeneration in adult *Xenopus* via the possible inhibition of histone decarboxylase (Taylor and Beck 2012). Therefore, this study investigated whether VPA affected caudal fin regeneration after amputation in adult zebrafish. For this purpose, we assessed the morphological alterations, such as outgrowth, segment, and bifurcation in the regenerated rays in the presence of VPA. Furthermore, we tested VPA's effect on cell proliferation and *lef1* expression in the regenerated ray at different times after amputation. Our study demonstrated that VPA exerted teratogenic effects during the fin regeneration and suppressed fin outgrowth.

## Materials and methods

### Fin amputation and drug treatment

Adult zebrafish purchased from a local fish shop were maintained at 28.0°C under a 14 h light-10 h dark cycle in an aquarium equipped with a continuous filtration and aeration system (Zebrafish Autosystem, Genomic Design, Seoul, Korea). Before the amputation of the caudal fin, zebrafish were anesthetized by immersion in water containing 0.2 mg/mL tricaine (Sigma-Aldrich, St. Louis, MO, USA). Immediately after amputation, zebrafish were treated with 20–200 μM of VPA (Sigma-Aldrich) for 1–14 days; the control group did not receive VPA treatment. The control and VPA-treated zebrafish were maintained at 32.0°C to facilitate fin regeneration after amputation.

### Alcian blue and alizarin red staining

Dermal fin rays were identified using Alcian blue and Alizarin red staining. First, the regenerated fins were fixed in 4% PFA overnight. After washing in phosphate buffer solution (PBS), the fins were bleached with 3% hydrogen peroxide and 1% KOH until the black pigments changed to a yellow color. Next, the fins were stained for 20 min at 37°C with filtered 0.3% Alcian blue in 30% acetic acid and 70% absolute ethanol. Afterward, they were dehydrated in a water–ethanol series for 3 min in each solution. Fins were then stored at 4°C in 100% ethanol overnight to fix the Alcian blue stain and de-stain the surrounding soft tissue. Fins were rehydrated in a water–ethanol series for 3 min in each solution and washed in running tap water for 15 min. After washing, the fins were stained with a filter solution of 1% Alizarin red S in 96% ethanol for 20 min at 37°C. The fins were washed again in running tap water for 15 min. Last, the stained fins were cleared by a graded KOH-glycerol series, 10 min each for 80:20 and 60:40, and kept at 4°C in 100% glycerol.

### Total RNA isolation and quantitative real-time PCR

The regenerated fins were flash-frozen in liquid nitrogen. Total RNA was extracted from 50 zebrafish fins at different days post-amputation (dpa) using the TRIzol reagent (Invitrogen, Carlsbad, CA, USA). The RNA was further purified with an RNeasy Mini Kit (Qiagen, Hilden, Germany). cDNA synthesis was performed using a superscript II cDNA synthesis kit (Invitrogen) with 2 μg of total RNA. For quantitative RT–PCR (qTR-PCR) analysis, 20-μl PCR reactions were freshly prepared on ice to include 12.6 μl of H_2_O, 2 μl of 10X universal buffer, 2 μl of 2.5 mM dNTP, 2 μl of primer at 10 pmol/μl, 0.2 μl of SYBR Green I, and 0.2 μl of Taq DNA polymerase. The unamputated adult fin was used as a control and β-actin as an internal control.

The following primers were used: *β-actin*, forward (5'-ATG GAT GAG GAA ATC GCT GCC-3’) and reverse (5'-CTC CCT GAT GTC TGG GTC GTC-3’); *wnt3a*, forward (5'-CCT TCT TCA AGC ATC CCA CTG-3’) and reverse (5'-TCT CTT TGC GCT TTT CTG TCC-3’); and *lef1*, forward (5'-GAG GGA AAA GAT CCA GGA AC-3’) and reverse (5'-AGG TTG AGA AGT CTA GCA GG -3’). The PCR conditions included a real-time extension for 5 min at 94°C, then 35 cycles of 1 min at 94°C, 1 min at 53°C, and 30 s at 72°C, followed by 2 min at 72°C for the final extension. The qTR-PCR was conducted in an ABI 7300 Real-Time PCR system (Applied Biosystem, Foster City, CA, USA). This procedure was repeated three times for each gene using three different experimental cDNA pools. The data's significance was evaluated *via* Student's *t*-test at a level of *p* < 0.05 level.

### Immunostaining for 5-bromo-2'-deoxyuridine

The zebrafish were anesthetized and treated with a single intraperitoneal injection of 10 mM 5-bromo-2'-deoxyuridine (BrdU) at 1 μL/100 mg body weight. BrdU was injected immediately after the zebrafish were terminated unless otherwise indicated. The fins were fixed in 4% paraformaldehyde with 0.01 M PBS at pH 7.4 and cryoprotected overnight with 30% sucrose at 4°C. The fins were cut into 14-μm cross-sections with a cryostat (CM1800; Leica, Wetzlar, Germany), mounted on coated slides (Matsunami Glass Ind., Ltd., Osaka, Japan), and stored at -20°C until future use. The sections were rinsed in 0.01 M PBS and incubated in 2 N HCl for 30 min at 37°C to denature the DNA. After blocking in 3% normal horse serum (Vector Laboratories, Burlingame, CA, USA) with 0.3% phosphate buffer saline with Triton X-100 (Sigma-Aldrich,), the sections were incubated overnight with mouse monoclonal anti-BrdU (DAKO; Glostrup, Denmark) at 4°C to label the S-phase cells. The next day, the sections were incubated with biotinylated horse anti-mouse IgG (Vector Laboratories) for 3 h at room temperature. The S-phase-labeled cells were then visualized using a diaminobenzidine substrate kit (Vector Laboratories) under a 20× objective lens using an Olympus BX50 microscope (Olympus America, Center Valley, PA, USA). The stained cells were counted in five consecutive sections to calculate the total number of stained cells in each regenerated fin.

## Results

### VPA suppresses outgrowth and altered fin formation

**Figure 1.**
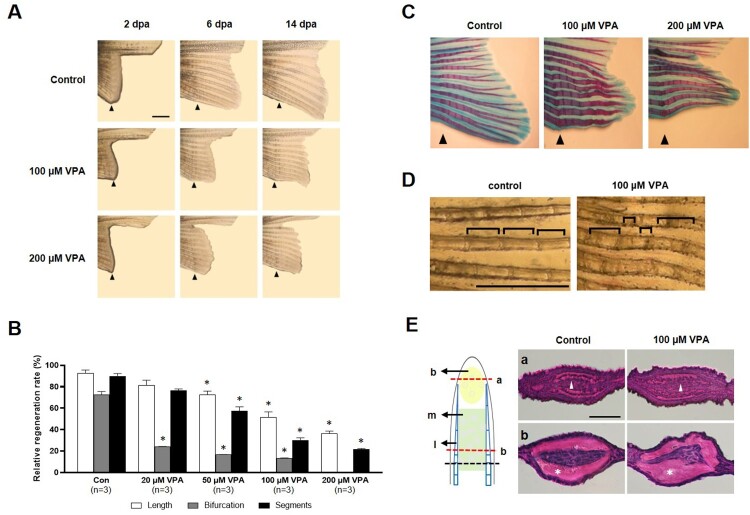
Suppression of caudal fin regeneration after amputation in VPA-treated zebrafish. (A) Images show the regenerated fin in each group at 2, 6, and 14 dpa. Arrowheads indicates the amputation site. Scale bar, 2 mm. (B) Bars indicate the regeneration ratio of length, segments, and bifurcating ray at 14 dpa. Data were expressed as the means ± S.E.M (*n *= 3). * *p* < 0.05 compared to the control. (C) Images show Alcian blue and alizarin red staining of cartilages and bones at 14 dpa. (D) Abnormal segments induced by 100 µM VPA treatment during regeneration. Scale bar, 1 mm. (E) Left: The regenerated intra-ray structure is schematically represented. A black dotted line, the amputation site. b, blastemia. m, mesenchyme. l, lepidotrichia. Right: Hematoxylin and eosin staining images show the transverse sections of intra-ray. The irregular-shaped lepidotrichia was shown in 100 µM VPA-treated zebrafish. Asterisks, bony skeletal structure. Arrow heads, actinotrichia. Scale bar, 500 µm.

First, the effects of VPA on fin regeneration was determined. The ventral caudal fin was partially amputated from the zebrafish and allowed to regenerate for 14 days in the presence of 20–200 μM VPA. Compared to the control, the outgrowth of the regenerated fin of VPA-treated zebrafish was suppressed in a dose-dependent manner [*F*(4, 10) = 11.77, *p* < 0.05, *n* = 3] (Figure 1A and B). At 14 days after amputation (dpa), the controls regenerated 92% of the fin's original length. In contrast, zebrafish treated with 20, 50, 100, and 200 μM VPA regenerated 82, 72, 55, and 40% of the original length, respectively. In general, zebrafish have 15–17 bifurcations in a ventral lobe of the caudal fin. At 14 days after dpa, the number of segments and bifurcating rays in the regenerated fin were also significantly reduced in a dose-dependent manner [segment; *F*(4, 10) = 42.33, *p* < 0.05, *n* = 3, bifurcating ray; *F*(4, 10) = 30.24, *p* < 0.05] (Figure 1B). In addition, VPA induced various malformations, such as fused bifurcating rays, thinner rays, and segments of inconsistent lengths (Figure 1C and D).

A transverse-section of the proximal part of the regenerated fin close to the amputated site showed a thick lepidotrichia without actinotrichia (Figure 1E). The lepidotrichia was smoothly curved and tapered along with the epidermis and mesenchymal area in the control. However, the lepidotrichia was irregularly bent in the zebrafish treated with 100 μM of VPA.

### The reduced number of BrdU-labeled cells in the regenerated fin of VPA-treated zebrafish

**Figure 2.**
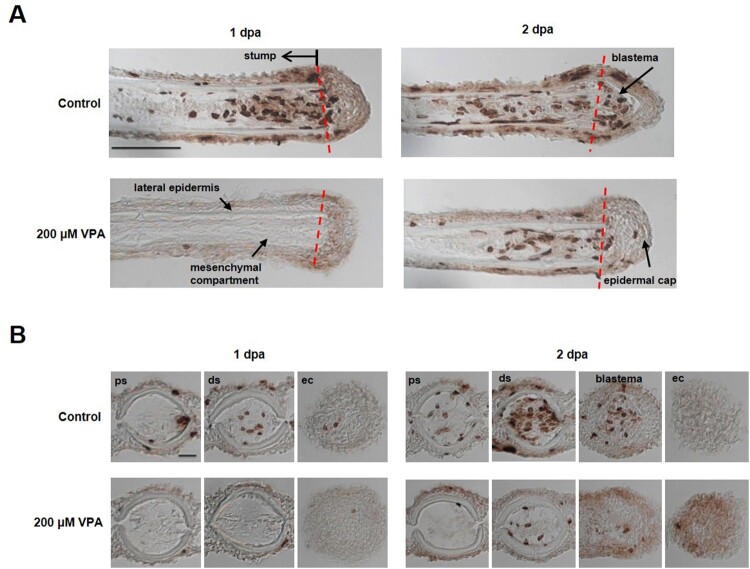
Delay of blastema formation in the regenerated fin by treatment with 200 µM VPA. (A) Images show the sagittal sections of the regenerated fin using BrdU staining at 1 and 2 dpa. A red dotted line, the amputation site. Scale bar, 500 µm. BrdU-labeled cells in the fin of control zebrafish were shown in the mesenchymal compartment, lateral epidermis and epidermal cap at 1 dpa, but not in 200 µM VPA-treated zebrafish. No blastema was formed in the regenerated area of 200 µM VPA-treated zebrafish. (B) At 1 dpa, BrdU-labeled cells were detected in the proximal stump (ps), distal stump (ds), and regenerated epidermal cap (ec) in the control, but hardly detected in 200 µM VPA-treated zebrafish. At 2 dpa, A few BrdU-labeled cells were detected in the distal stump of 200 µM VPA-treated zebrafish. Scale bar, 200 µm.

Since cell proliferation is expected to increase during fin regeneration, the number of BrdU-labeled, S-phase cells in the regenerated fin was measured at 1 and 2 dpa. At 1 dpa, the sagittal section of the regenerated fin of the controls showed a newly developed wound epidermis above the amputated site; BrdU-labeled cells were found in the stump but not in the wound epidermis (Figure 2A). On the other hand, BrdU-labeled cells were rarely detected in the zebrafish’ stump treated with 200 μM VPA. At the same time, the cross-sections also showed that BrdU-labeled cells were found in the control stump, but not in the stump of the zebrafish treated with 200 μM VPA (Figure 2B).

At 2 dpa, the blastema was developed in the controls, and BrdU-labeled cells were found in the blastema as well as in the stump of the control (Figure 2A). In contrast, the blastema was not yet developed in the regenerated fin of the zebrafish treated with 200 μM VPA; but BrdU-labeled cells began to appear in the stump (Figure 2A). At the same time, BrdU-labeled cells were found in the control's stump and blastema but only in the zebrafish’ stump treated with 200 μM VPA (Figure 2B).

**Figure 3.**
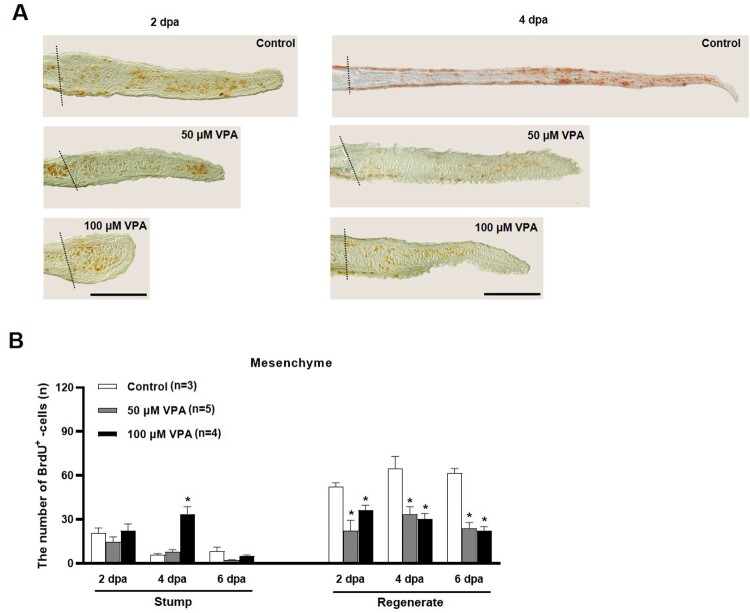
Quantification of BrdU-labeled cells in the mesenchymal area of regenerated fin at 2, 4, and 6 dpa. (A) Images show BrdU-labeled cells in the sagittal sections at 2 and 4 dpa. A dotted line indicates the amputation site, and brown colored dots are BrdU-labeled cells. Scale bar = 500 µm. (B) Bars indicate the number of BrdU-labeled cells in mesenchymal area of the stump and regenerated fin at 2, 4, and 6 dpa. Data were expressed as the means ± S.E.M (n≥3) and tested via *post hoc* Tukey's multiple comparison tests. * *p* < 0.05 compared to the control.

The number of BrdU-labeled cells was compared in the regenerated fin to further evaluate cell proliferation during fin regeneration. At 2 and 4 dpa, the regenerated fin's outgrowth in the control group was longer than those in the zebrafish treated with 50 and 100 μM VPA (Figure 3A). Also, the numbers of BrdU-labeled cells in the mesenchymal compartment of the regenerated fin were significantly low in the VPA-treated zebrafish than the control (Figure 3B).

### VPA does not increase the lef1 mRNA level in the regenerated fin

**Figure 4.**
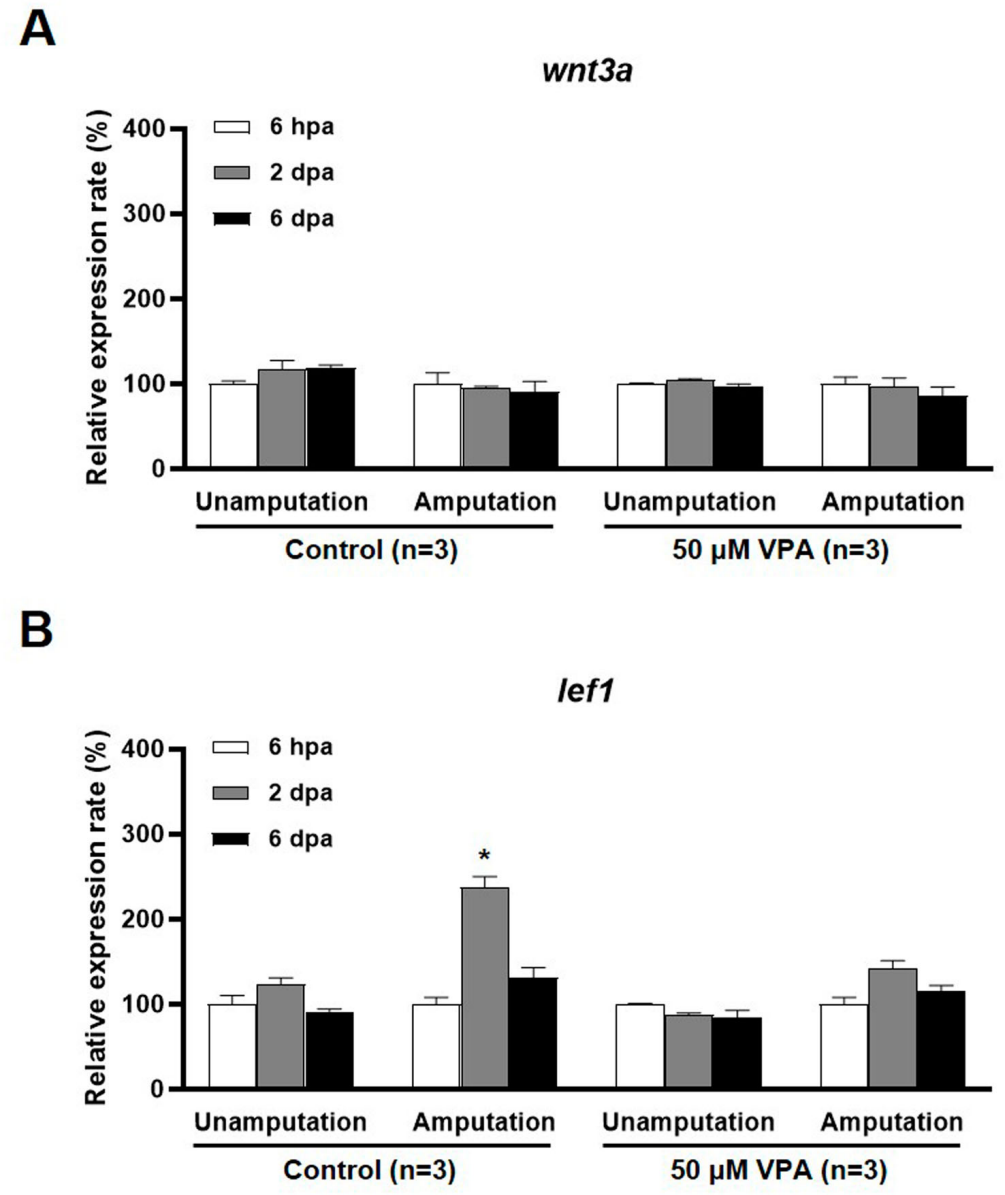
qRT-PCR analysis of *wnt3a* and *lef1* mRNA expression in the regenerated fin at 6 hpa, 2 and 6 dpa. Bars indicate the expression levels of *wnt3a* and *lef1* mRNA in the control and 50 µM VPA-treated zebrafish fin. (A) No significant change in *wnt3a* mRNA expression was observed in both the control and 50 µM VPA-treated zebrafish. (B) Expression levels of *lef1* mRNA increased only in regenerated fin of control at 2 dpa. The experiments were repeated 3 times. Data were expressed as the means ± S.E.M and were analyzed via *post hoc* Tukey's multiple comparison tests. * *p* < 0.05 compared to the 6 hpa.

The Wnt signaling pathway is suggested to be involved in the cell proliferation, skeletal development, and the tissue regeneration (Hartmann 2007; Zhang et al. 2019; Raslan and Yoon 2020). VPA's effect on the expression of *wnt3a*, which encodes a Fz receptor agonist, and *lef1*, which encodes a downstream signaling molecule in the Wnt pathway, during fin regeneration was examined. The level of *wnt3a* mRNA was not altered at 6 h post amputation (hpa), 2, and 6 dpa in the VPA treatment or the control group (Figure 4A). However, at 2 dpa, the level of *lef1* mRNA was significantly increased in the control but remained notably unchanged in the regenerated fin of the zebrafish treated with 50 μM VPA [*F*(2, 6) = 56.03, *p* < 0.05, *n* = 3] (Figure 4B).

### Sodium butyrate suppresses fin regeneration

**Figure 5.**
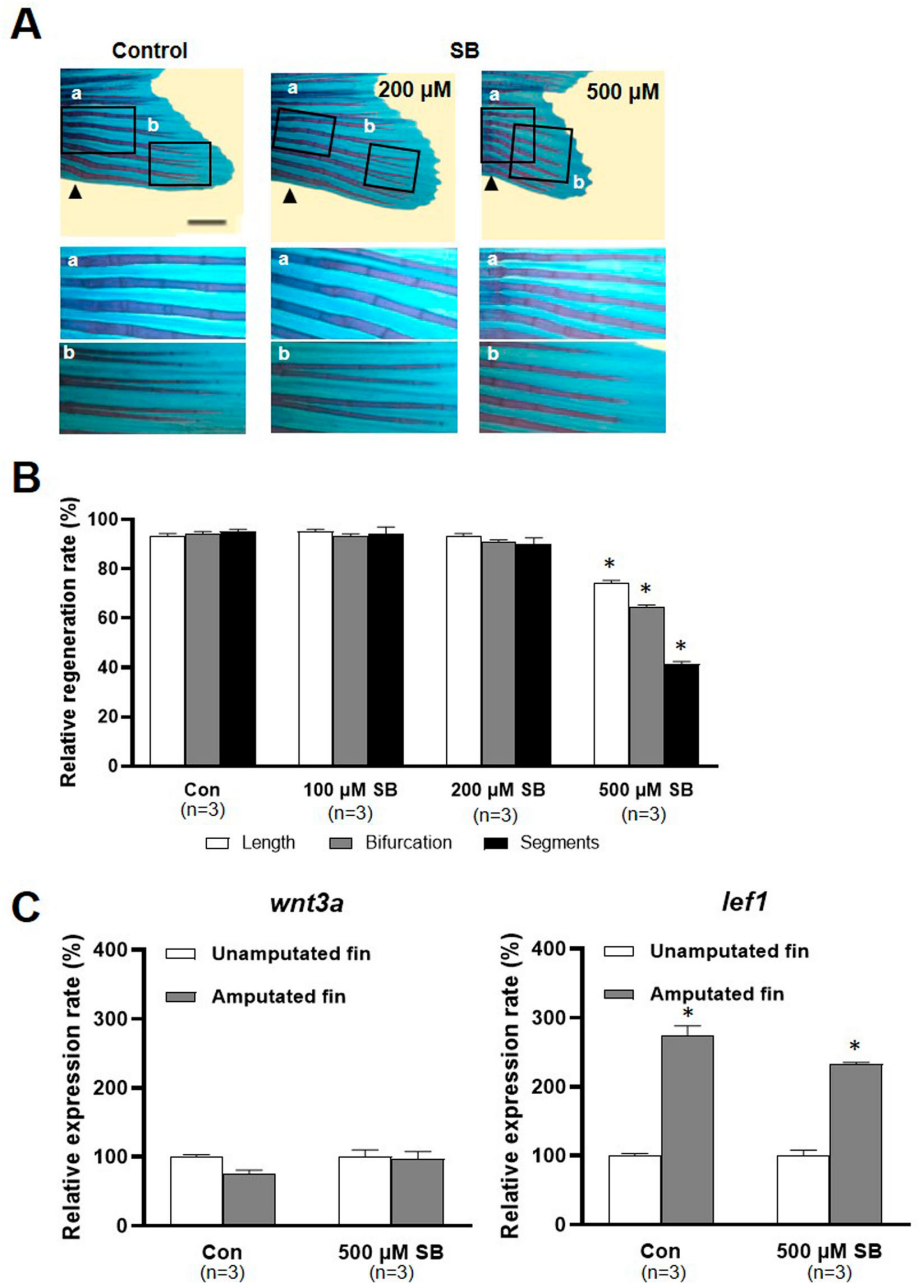
Effects of sodium butyrate on caudal fin regeneration of adult zebrafish. (A) Images show Alcian blue and alizarin red staining of the regenerated fin in the control and SB (200 and 500 µM)-treated zebrafish at 14 dpa. Arrowheads, the amputation site. Scale bar, 2 mm. (B) Bars indicate the regeneration ratio of length, segments, and bifurcating ray at 14 dpa. Decreased regeneration rate was shown in only 500 µM SB-treated zebrafish. Data were expressed as the means ± S.E.M (*n *= 3). * *p* < 0.05 compared to the control. (C) Bars indicate the expression levels of *wnt3a* and *lef1* mRNA in 500 µM SB-treated zebrafish fin at 2 dpa compared to control. Expression levels of *lef1* mRNA increased in both control and SB-treated zebrafish at 2 dpa. The experiments were repeated 3 times. Data were expressed as the means ± S.E.M and were analyzed with Student’s *t*-tests. * *p* < 0.05 compared to the unamputated fin.

VPA is known to inhibit histone deacetylases (HDACs). The effects of sodium butyrate (SB), an HDAC inhibitor, on fin regeneration were compared to those of VPA. Little structural malformation was found in the regenerated fin of SB-treated zebrafish (Figure 5A). Compared to the control, the length of the regenerated fin the zebrafish treated with 100 and 200 μM SB was not shorter (Figure 5A and B). Neither the number of segments nor bifurcating rays in the regenerated fin in the zebrafish treated with 100 and 200 μM SB was different from those in the control (Figure 5B). In contrast, at 14 dpa, the length of the regenerated fin, the number of segments, and bifurcating rays were significantly reduced in zebrafish treated with 500 μM SB compared to the control (Figure 5B).

SB's effect on the Wnt signaling pathway was also studied. At 2 dpa, the wnt3a mRNA level was not changed in the SB treatment group or the control group. Meanwhile, at 2 dpa, the level of *lef1* mRNA was significantly increased in the control and the zebrafish treated with 500 μM SB (*p* < 0.05, *n *= 3), in contrast to the lack of effect of VPA on the level of *lef1* (*p* > 0.05, *n* = 3; Figure 5C).

### The malformation of the regenerated fin induced IWR-1, a Wnt antagonist

Since VPA suppressed the increased level of *lef1* mRNA during fin regeneration, the effects of IWR-1, a Wnt antagonist, on fin regeneration were studied. The length of the regenerated fin was slightly short in the 500 nM IWR1-treated zebrafish compared to the control (Figure S1A and B), but the regenerated rays in the IWR-1 group appeared irregularly bent and thinner (Figure S1A). At 14 dpa, the length of regenerated fin was shorter in the zebrafish treated with 50 μM VPA than those in the control [*F*(4, 20) = 20.23, *p* < 0.05, *n* = 5] (Figure S1B). Also, the ratio of the number of segments and bifurcating rays in the regenerated fin was decreased in both VPA- and IWR1-treated zebrafish [segment; *F*(4, 20) = 49.07, *p* < 0.001, *n* = 5, bifurcating ray; *F*(4, 20) = 11.45, *p* < 0.001, *n* = 5] (Figure S1B).

## Discussion

This study showed that the amputated caudal fin of adult zebrafish was regenerated to its original length and pattern at 14 days after amputation. Meanwhile, the treatment with VPA immediately after amputation not only suppressed the regrowth of caudal fin in length but also altered the formation of rays and segments, causing malformations, such as reduced thickness and abnormal bending of ray.

Several types of malformations occurred following VPA exposure during pregnancy. Mainly, VPA treatment causes skeletal malformations, such as the fusion of vertebrae and ribs and the duplication of some segments during the embryonic stage (Massa et al. 2005; Menegola et al. 2005). However, these studies were performed using high doses of VPA between 400 and 600 mg/kg; 500 mg of VPA dissolved in 1 L of water is calculated to be 2.6 mM, which is significantly higher than the dose range of 20–200 µM VPA used in our study. As an anti-epileptic drug, VPA's therapeutic dose ranges between 50 and 100 µg/mL, or 294–588 µM in the patient's serum (Kanner 2003). Notably, our study showed that even a bath treatment with VPA at a dose below the therapeutic range could cause the malformation of the regenerated caudal fin of adult zebrafish.

According to previous studies, VPA suppresses cell proliferation, delays cell cycle, and induces apoptosis *in vitro* and *in vivo* (Michaelis et al. 2004; Chen et al. 2006; Lee et al. 2014; Zhu et al. 2015). Our study showed that, compared to the control, the cell proliferation in the mesenchymal compartment of the regenerated caudal fin was significantly decreased in VPA-treated zebrafish. Blastema formation was also delayed in VPA-treated zebrafish at 2 days after amputation compared to the control. Therefore, suppressing the regeneration of amputated caudal fins may decrease the proliferation of scleroblasts and osteoblasts, which are involved in regeneration.

VPA is known to inhibit HDAC in zebrafish and *Xenopus* embryos (Gurvich et al. 2005; Taylor and Beck 2012). It has been reported that 50–150 µM VPA treatment for 24–48 h decreases the number of regenerated hair cells in the lateral line of zebrafish larvae in a dose-dependent manner (He et al. 2014). This finding was partly supported by the observed suppressive effects of SB, another HDAC inhibitor, on fin regeneration. Our study showed that the regeneration rate was decreased in zebrafish treated with 500 μM SB compared to the control. However, the malformation of the regenerated fin was less extensive in SB-treated zebrafish than in VPA-treated zebrafish. Therefore, VPA treatment likely inhibits Wnt signaling during fin regeneration and blocks cell proliferation. Furthermore, the treatment of IWR-1, which inhibits Wnt signaling, results in malformations similar to those observed in VPA-treated zebrafish.

The differential effects of VPA and SB on fin regeneration suggest that VPA's effect would not be entirely attributable to the inhibition of HDAC (Wiltse 2005). This study has shown that expression of *lef1* mRNA, a downstream molecule of the Wnt signaling pathway, was increased in the regenerated fin of the control (Poss et al. 2000) and the SB-treated zebrafish at 2 dpa, but not in the VPA-treated zebrafish. Therefore, the effects of VPA below the therapeutic range on fin regeneration may be mediated by reducing cell proliferation and blocking the Wnt signaling pathway.
